# Optogenetic control of early embryos labeling using photoactivatable Cre recombinase 3.0

**DOI:** 10.1002/2211-5463.13862

**Published:** 2024-09-02

**Authors:** Kumi Morikawa, Akira Nagasaki, Lue Sun, Eihachiro Kawase, Tatsuhiko Ebihara, Yasuaki Shirayoshi

**Affiliations:** ^1^ Cellular and Molecular Biotechnology Research Institute National Institute of Advanced Industrial Science and Technology (AIST) Tsukuba Japan; ^2^ Biomedical Research Institute National Institute of Advanced Industrial Science and Technology (AIST) Tsukuba Japan; ^3^ Health and Medical Research Institute National Institute of Advanced Industrial Science and Technology (AIST) Tsukuba Japan; ^4^ Institute for Life and Medical Sciences Kyoto University Kyoto Japan; ^5^ Division of Regenerative Medicine and Therapeutics, Department of Genomic Medicine and Regenerative Therapy, Faculty of Medicine Tottori University Yonago Japan

**Keywords:** Cre‐*loxP* recombination, early embryos, lineage tracing, optical regulation, optogenetics, photoactivatable Cre recombinase

## Abstract

Establishing a highly efficient photoactivatable Cre recombinase PA‐Cre3.0 can allow spatiotemporal control of Cre recombinase activity. This technique may help to elucidate cell lineages, as well as facilitate gene and cell function analysis during development. This study examined the blue light‐mediated optical regulation of Cre‐*loxP* recombination using PA‐Cre3.0 transgenic early mouse pre‐implantation embryos. We found that inducing PA‐Cre3.0 expression in the heterozygous state did not show detectable recombination activation with blue light. Conversely, in homozygous embryos, DNA recombination by PA‐Cre3.0 was successfully induced by blue light and resulted in the activation of the red fluorescent protein reporter gene, while almost no leaks of Cre recombination activity were detected in embryos without light illumination. Thus, we characterize the conditions under which the PA‐Cre3.0 system functions efficiently in early mouse embryos. These results are expected to provide a new optogenetic tool for certain biological studies, such as developmental process analysis and lineage tracing in early mouse embryos.

## Graphical abstract

Optogenetic techniques enable spatiotemporal gene expression control via light illumination, which is precisely regulated at the organelle level and within millisecond time frames. In this study, we used Photoactivatable Cre recombinase 3.0 (PA‐Cre3.0) for light‐induced Cre‐*loxP* recombination and identified conditions conducive to light‐induced Cre‐*loxP* recombination in early mouse embryos. Our findings demonstrate the applicability of PA‐Cre3.0 for developmental lineage tracing.

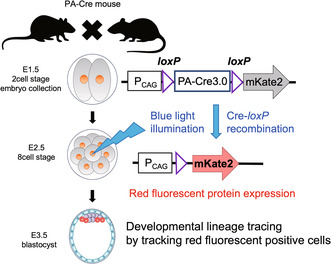

**Abbreviations**
hCG: human chorionic gonadotropin
MEFs: mouse embryonic fibroblasts
PMSG: Pregnant mare serum gonadotropin

DNA recombination is widely used to regulate gene expression in various fields, such as genetics, cell biology, and embryology. One such method is Cre‐*loxP* recombination, in which the Cre recombinase induces a reaction between loxP sequences [1, 2]. The development of this method has focused on achieving spatiotemporal control of gene expression with utilization of tissue‐ and cell‐specific enhancers and tamoxifen‐induced Cre‐estrogen receptors [3]. However, the current Cre‐*loxP* system lacks precision in spatiotemporal regulation, and the use of chemicals has limitations in terms of cytotoxicity and administration methods to the target site [4], making it a barrier to accurate analysis of gene function by recombination.

Recently, tools for controlling gene expression using optogenetics have been developed, enabling spatiotemporal control of gene expression, and providing new insights into biological functions [5]. One such tool is the photoactivatable Cre (PA‐Cre) recombinase, which was developed to control the enzymatic activity of Cre recombinase in a spatiotemporal manner. Two types of PA‐Cre, the CRY‐CIBI and Magnets systems, have been previously reported [6]. The former is based on the introduction of mutations into CRY2 to an improved PA‐Cre2.0 [7], while the latter utilizes the more efficient photodimerization tool, Magnets [8]. This Magnets system modifies the photoreceptor Vivid [9, 10], leading to the complementation of the Split‐Cre protein and activation of the Cre protein. The activated Cre protein recognizes the *loxP* sequence and induces recombination between the *loxP* sites. The Magnets system can further cause the knockout of the gene region flanked by *loxP* sequences or induce the expression of downstream genes (Fig. S1). Recently, the Magnets‐based system has evolved into PA‐Cre 3.0, which is highly efficient in inducing recombination through blue light and reducing leakage on darkening by optimizing protein expression levels [11]. It combines the de‐optimization of amino acid codons to reduce the expression of the PA‐Cre protein. The promoter of PA‐Cre regulation is also changed to a CAG promoter, which is expected to be ubiquitously expressed *in vivo* [12].

Analyses using optogenetic tools have mainly been conducted in cranial nerves [13].

Regarding light control of Cre‐*loxP* recombination, *in vivo* tissues such as heart, lungs, and kidneys encounter issues with light transmissibility. The permeability of blue light is limited to a maximum of 2–3 mm *in vivo*, which is a major limitation for light induction of recombination and subsequent observation of reporter fluorescence. Furthermore, in mammals, the developmental process proceeds in utero, making it difficult to target cells or tissues with pinpoint accuracy by light. For these reasons, it isn't easy to validate the performance of PA‐Cre3.0 in mammalian biological tissue, especially about precise spatiotemporal control by light. Early embryos as the subject of analysis are easier to handle than tissues, and if the technology can be well controlled, they could be a good analysis system. It is, therefore, considered one of the ideal experimental systems for confirming the utility of precise spatiotemporal analysis of the photo regulation of Cre‐*loxP* recombination.

Multiple transgenic mouse models of Magnets‐based PA‐Cre have been developed [11, 14, 15], but few studies have examined their utility in early development [11]. In addition, in the early embryo studies of PA‐Cre transgenic mice, the efficiency of Cre‐*loxP* recombination with blue light was very low, so stronger blue light exposure or stronger expression of the PA‐Cre enzyme has been advocated [11].

This study aims to address a technical gap that the existing PA‐Cre system has in the analysis of biological phenomena during embryogenesis. Recently, transgenic mice introducing an improved PA‐Cre3.0 expression vector at Rosa26 locus (A20 line) have been developed [12]. Using various transgenic mice related to Cre‐*loxP* recombination including A20 line, we explore for the conditions under which Cre‐*loxP* recombination is efficiently regulated by light in early mouse embryos. The results show that light‐responsive Cre recombination functions well by homogenizing the ROSA26 locus (Rosa26^PA‐Cre A20/WT^) in A20 mice. Thus, the increased expression of PA‐Cre3.0 in A20 homozygous mice may provide a precise optical control system for Cre‐*loxP* recombination, indicating the utility of improved PA‐Cre3.0 in embryogenesis analysis.

## Materials and methods

### Animals

Animal experiments were performed in accordance with the Guidelines for the Care and Use of Laboratory Animals of the Animal Committee of the National Institute of Industrial Science and Technology (AIST) (approval numbers A2022‐0374‐A and A2021‐0374). Rosa^PA^ ^‐Cre A20^ (B6;129S‐*Gt (ROSA)26Sor* ^tm1(CAG‐cr*,‐mKate2) Yzwa^/J: #033544) and Rosa^PA^ ^‐Cre B4^ (B6;129S‐*Gt (ROSA)26Sor* ^tm2(CAG‐cre*) Yzwa^/J: #033545) were obtained from The Jackson Laboratory (Bar Harbor, ME, USA). R26GRR (C57BL/6N‐Gt (ROSA)26Sor<tm1(CAG‐EGFP/tDsRed) Utr>/Rbrc: RBRC04874) and CAG‐Flpe (C57BL/6‐Tg (CAG‐flpe)36Ito/ItoRbrc: RBRC01834) mice were purchased from RIKEN BRC (Ibaraki, Japan). To maintain the mouse strains and for mouse embryonic fibroblast (MEF) experiments, the following primer sets were used for genotyping PCR: Rosa^PA^ ^‐Cre A20^ and Rosa^PA^ ^‐Cre B4^: forward 5′‐GGACTAGGGCTGCGTGAGTCTCTGA‐3′, reverse 5′‐GGCGTTACTATGGGAACATACGTC‐3′; CAG‐Flpe: forward 5′‐CCTACAGCTCCTGGGCAACGTGC‐3′, reverse 5′‐CTGCTTCTTCCGATGATTCG‐3′. The primers for detecting the stop sequence excision were as follows: forward, 5′‐CTCTGCTAACCATGTTCATGCCTTCTTC‐3′, and reverse, 5′‐CAGAGTATAAGGGCGCAAGAAGTGTCAA‐3′ (Oligo Synthesis, IDT, Singapore, Singapore).

### Embryo isolation

Ten units of pregnant mare serum gonadotropin (PMSG) (Asuka Pharmaceutical Co., Tokyo, Japan) and human chorionic gonadotropin (hCG) (Asuka Pharmaceutical Co.) were injected intraperitoneally into female mice 4 and 2 days before embryo collection, respectively. E1.5 embryos were collected from the oviduct using a 30G syringe and flushed. Isolated embryos were washed with FHM medium (Sigma‐Aldrich, Burlington, MA, USA) three times and cultured in KSOM medium (Kyudo Company, Tosu, Japan) at 37 °C, 5% CO_2_.

### Mouse embryonic fibroblast isolation

Mouse embryonic fibroblasts (MEFs) were isolated from Rosa^PA‐Cre A20^ heterozygous female mice mated with CAG‐Flpe heterozygous male mice. E10.5 embryos were isolated and washed with PBS four times. Then, each embryo was treated with 0.25% trypsin–EDTA (Wako Pure Chemical Co., Osaka, Japan) at 37 °C for 40 min. MEFs were then cultured in DMEM (Sigma‐Aldrich) supplemented with 10% fetal bovine serum (Nichirei Biosciences Inc., Tokyo, Japan), 1% pyruvate (Thermo‐Fisher Scientific Inc., MA, USA), 1% GlutaMax I (Thermo‐Fisher), and penicillin–streptomycin (Wako, Osaka, Japan) under normoxia (20% O_2_, 5% CO_2_, 37 °C) using a CO_2_ incubator (SANYO Electric Co., Ltd., Osaka, Japan). Residual embryo tissue was used for genomic DNA extraction for genotyping PCR analysis.

### Blue light illumination

Mouse embryos and MEFs were illuminated with an LED source (470 ± 20 nm, 2 W·m^−2^; CCS Inc., Kyoto, Japan) under normoxia. The embryos and the MEFs were continuously illuminated for 24 and 6 h, respectively. After illumination, the cells were kept in the dark and imaged for fluorescence reporter expression. We used the following instruments to capture MEFs images. Microscope; Nikon ECLIPSE Ti2R, Camera; Mono Nikon DS‐Ri2, Software: nis‐elements br 5.10.00 64‐bit, Objective lens: Nikon S Plan Fluor ELWD 20×/0.45, Objective magnification: 20×, Bining: 1.0 × 1.0, Exposure: 1 s, Gain: 64.0×, Fluorescence Filter: TRITC, Emission wavelength: 535.0. We used following instruments to take embryos images. Microscope; Olympus IX73, Camera; Olympus DP74, Software: cellsens standard ver.1.17, Objective lenses: Olympus LUCPlan FLN 20×/0.45 (low magnification images) and Olympus LUCPlan FLN 40×/0.60 (high magnification images), Objective magnification: 20× (low magnification images) and 40× (high magnification images), Bining: 1.0 × 1.0, Exposure: 1–5 s, Gain: 0.50×, Mirror Unit: U‐FGFP, Fluorescence Filter: GFP and RFP, Light source: Olympus U‐RFL‐T. Images were analyzed using imagej software version 2.0.0‐rc‐59/1.51n (NIH, Bethesda, MD, USA).

## Results

### Overview of the PA‐Cre system and how it works in transgenic mice

**Fig. 1:**
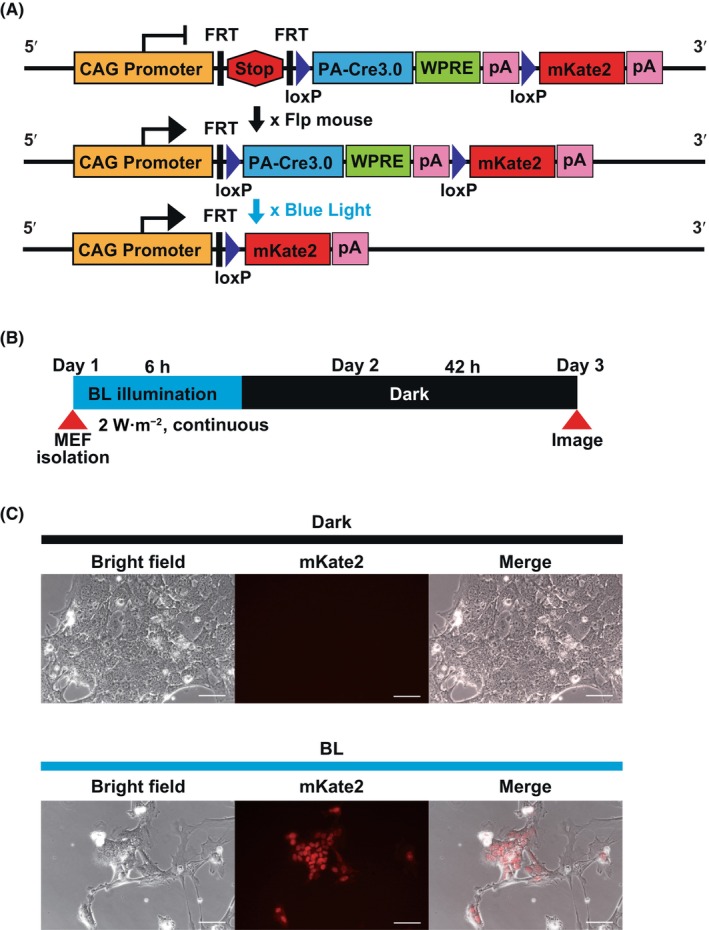
Generation and validation of an all‐in‐one type of PA‐Cre3.0. (A) Schematic representation of the all‐in‐one type of PA‐Cre3.0 mouse targeting strategy. Construction of the all‐in‐one type targeting vector for PA‐Cre3.0 and its reporter at the ROSA26 locus (top). PA‐Cre3.0 expression is induced after the polyadenylation transcriptional terminator “stop” signal is deleted via Flp‐mediated recombination (middle). Blue light activation of PA‐Cre3.0 recombinase results in mKate2 reporter expression in the nucleus through the Cre‐mediated self‐deletion of the PA‐Cre3.0 expression cassette (bottom). (B) Experimental protocol for mouse embryonic fibroblasts (MEFs) prepared from A20 mice. (C) Biofluorescence images of mKate2 in MEFs of A20:CAG‐Flpe F1 fetuses. MEFs isolated from E10.5 fetuses were cultured with or without blue light illumination for 6 h (Blue LED, 447 nm, 2 W·m^−2^, continuous), followed by 42 h in the dark and observation under fluorescence microscopy. MEFs from embryo #2 (Fig. S2/lane #2) were used for imaging. Scale bar = 50 μm, *n* = 2.

Two transgenic mouse lines, A20 and B4, were used in this study. The former has an all‐in‐one type of pCAG‐PA‐Cre3.0 expression vector knocked in the ROSA26 locus with the reporter gene of mKate2 red fluorescent protein [16], whereas the latter is a simplified line containing only pCAG‐PA‐Cre3.0. In the A20 all‐in‐one vector (Fig. 1A), the polyadenylation transcriptional terminator “stop” (poly‐A) signal was sandwiched between the *FRT* cassette and inserted between the promoter and the enzyme. Furthermore, PA‐Cre3.0 was flanked by a *loxP* cassette and located upstream of mKate2. Thus, under normal conditions, the stop poly‐A signal suppresses the expression of both PA‐Cre3.0 and mKate2. Crossing with flippase (Flp) mice led to the loss of the stop poly‐A signal and expression of PA‐Cre3.0 under the control of the CAG promoter.

Blue light‐activated PA‐Cre proteins recognize *loxP* sequences and undergo self‐recombination. This recombination removes both the PA‐Cre3.0 cassette body and the poly‐A signal, which induces the expression of a red fluorescent protein reporter. Thus, in the A20 all‐in‐one system, PA‐Cre3.0 is temporally activated by blue light illumination, and the recombinant cells can be labeled with red fluorescence. In contrast, B4 mice lack the red fluorescent reporter cassette and are not equipped with *loxP*‐based self‐recombination elements. Therefore, in the B4 line, the Flp‐mediated excision of the *FRT*‐Stop‐*FRT* cassette led to the continuous expression of PA‐Cre3.0.

### Verification of light‐induced recombination in MEFs derived from A20 mice

Because we used a Flp mouse line different from previous studies [12, 17], we first examined whether the all‐in‐one type of system of PA‐Cre3.0 of the A20 line is activated using blue light. A20 mice (Rosa^PA‐Cre A20/WT^: heterozygous) were crossed with CAG‐Flpe mice (heterozygous), and MEFs were prepared from F1 mouse fetuses at E10.5, as previously reported [12]. The prepared MEFs were examined using genomic PCR to determine whether the stop signal was normally removed by Flp‐*FRT* recombination (Fig. S2). The results showed that stop signal removal occurred in MEFs from embryos carrying PA‐Cre3.0 and CAG‐Flpe. In contrast, no stop signal removal was detected in MEFs derived from fetuses carrying either CAG‐Flpe or PA‐Cre3.0. Thus, we confirmed that Flp‐*FRT* recombination by mating caused stop signal removal. We then examined whether the expression of PA‐Cre3.0 was induced after stop signal removal and activated in a blue light‐dependent manner. Exposure of cultured MEFs to blue light at an intensity of 2 W·m^−2^ for 6 h resulted in the expression of mKate2 in the nucleus (Fig. 1B,C). In the A20 line, after mating with Flp mice, the activation of PA‐Cre3.0 and expression of the mKate2 reporter were induced by blue light, as designed.

### Investigation of light‐operated gene recombination in early embryos using the A20 line

**Fig. 2:**
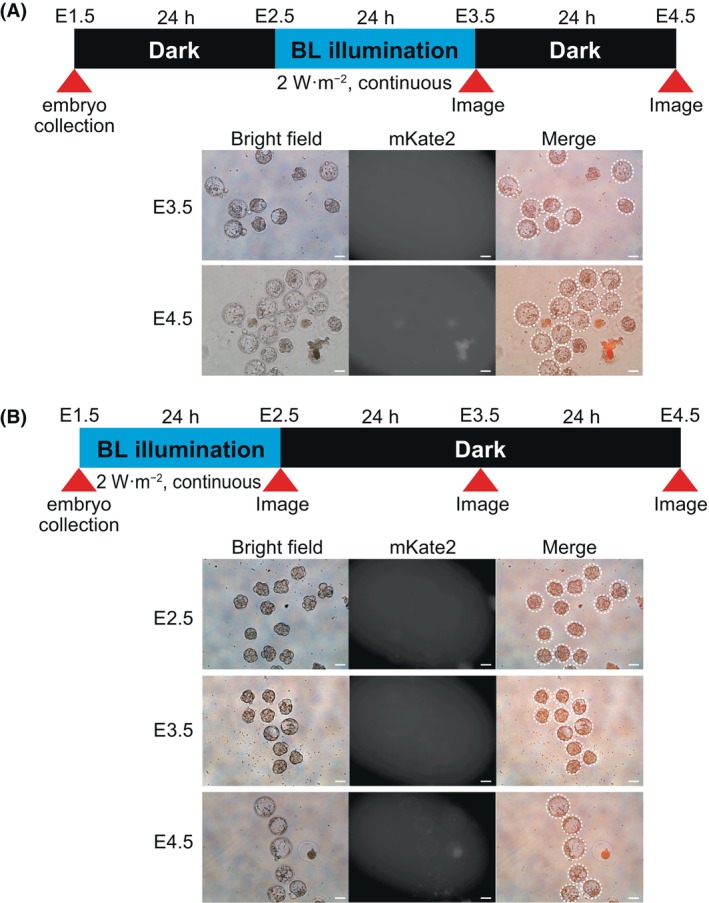
Blue light‐induced photoactivation in A20 mouse embryos. (A) Photoactivation assay for PA‐Cre3.0 in the A20 line. The upper diagram shows the experimental protocol to confirm the photoactivation of PA‐Cre3.0. Heterozygous A20 mice were mated with heterozygous CAG‐Flpe mice. F1 embryos were collected at E1.5 and cultured for 1 day in the dark, followed by 24 h blue light illumination at the same conditions as in Fig. 1B. mKate2 fluorescence was examined using fluorescence microscopy at E3.5 and E4.5. Abnormal embryos showed red auto‐fluorescence. Scale bar = 50 μm, *n* = 3. (B) Schedules of the different assays for photoactivation of PA‐Cre3.0 in A20 line mice. The upper diagram shows another experimental protocol. The collected F1 embryos at E1.5 were directly illuminated under blue light for 24 h with the same conditions as in Fig. 1B. At E2.5, E3.5, and E4.5, mKate2 fluorescence was examined using fluorescence microscopy. The normal developmental embryos were circled with white dashed lines. Scale bar = 50 μm, *n* = 1.

We then tested whether the all‐in‐one type of PA‐Cre3.0 system of the A20 line could label cells of early embryos in the same manner as MEFs. Detecting of mKate2 reporter fluorescence requires several processes, including Cre‐*loxP* recombination, transcriptional activation, and subsequent mRNA translation. Therefore, considering the time required for these processes, the timing for observing mKate2 reporter fluorescence was set at 24 or 48 h after blue light illumination. To keep embryo collection and observation timing constant, we used two protocols with blue light illumination at E1.5–2.5 and E2.5–3.5, as shown in Fig. 2A,B. These two timings are also linked to the major activation of the zygotic genome in the maternal‐to‐zygotic transition: at E1.5, the embryo is at the 2‐cell stage when zygotic genes are activated, and maternal factors direct development. At E2.5, on the other hand, the embryo reaches the 8‐cell stage, and developmental control shifts to the germ nucleus [18].

Fertilized eggs were collected from F1 mice between A20 and CAG‐Flp mice and exposed to blue light at 2 W·m^−2^ for 24 h from E2.5–E3.5. No early embryos showed detectable induction of mKate2 expression upon Cre*‐loxP* recombination (Fig. 2A) at E4.5. We next attempted a similar experiment by changing the timing of the blue light illumination at E1.5‐E2.5. During egg retrieval, embryos were illuminated with blue light at an intensity of 2 W·m^−2^ for 24 h from E1.5–E2.5, and mKate2 expression was examined 24 and 48 h later. None of the early embryos had visibly induced mKate2 expression in the nucleus by Cre‐*loxP* recombination (Fig. 2B). Only weak auto‐fluorescence was observed in embryos that had arrested development, and no embryos expressing mKate2 were detected. In the F1 embryos from CAG‐Flpe heterozygous and A20 heterozygous mice, the removal of the stop signal upon PA‐Cre3.0 activation by blue light was expected to occur with a probability of 1/4, but mKate2 expression was not induced by blue light in any of the embryos collected. Because detectable mKate2 expression was not observed in early mouse embryos, we considered that PA‐Cre3.0 enzyme activity was too low to induce Cre‐*loxP* recombination or that mKate2 fluorescence was weak and insufficient to detect, albeit induction of DNA recombination. We proceeded with the further investigation as follows.

### Cre‐ *loxP* recombination was lacking in B4‐crossbred embryos

**Fig. 3:**
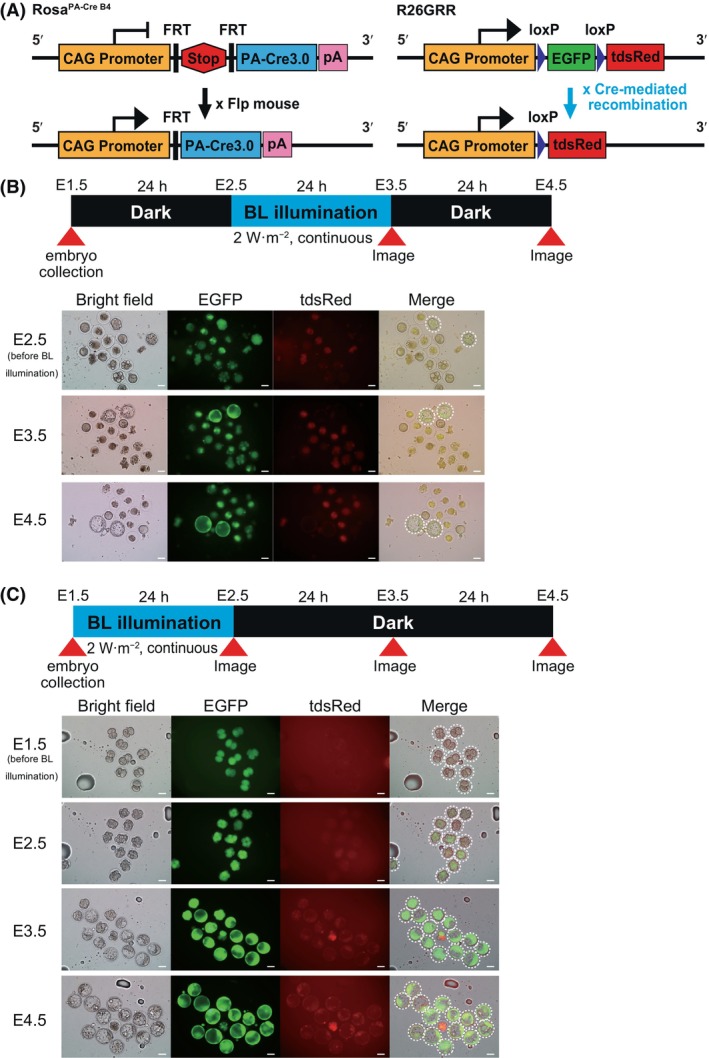
Validation of a simple version of PA‐Cre3.0 in B4 mouse embryos. (A) Schematic representation of the B4 version of PA‐Cre3.0 and the R26GRR reporter strain. Crossing with Flp mice results in the induction of PA‐Cre3.0 expression in B4 mice. Photoactivated PA‐Cre3.0 causes tdsRed expression instead of EGFP expression in R26GRR mice. (B) Experimental protocol to confirm the successful photoactivation of PA‐Cre3.0. The same schedule for blue light illumination as in Fig. 1C was applied for F1 embryos obtained by crossing heterozygous B4 and homozygous R26GRR mice. Scale bar = 50 μm, *n* = 1. (C) Fluorescence images of F1 embryos at E1.5–E4.5. All F1 embryos did not show tdsRed fluorescence but showed EGFP fluorescence with significant red auto‐fluorescence. Abnormal embryos showed strong red auto‐fluorescence. The normal developmental embryos were covered with white dashed lines. Scale bar = 50 μm, *n* = 1.

Because previous studies have shown that the A20 line has a relatively weak expression of the reporter mKate2 [12], we decided to use R26GRR mice (Fig. 3A) [19], which have strong reporter fluorescence expression, to label recombinant cells with PA‐Cre. In these mice, recombination converted EGFP expression into tdsRed expression. In anticipation of stronger recombination activity owing to the permanent expression of PA‐Cre, the B4 line, a simpler version of the A20 mouse line, was used (Fig. 3A).

E2.5 fertilized eggs obtained by crossing F1 mice of the B4 strain (Rosa^PA‐Cre B4/WT^: heterozygous) and CAG‐Flpe mice (CAG‐Flpe^+/WT^: heterozygous) with R26GRR homozygous mice were collected and then exposed to blue light at 2 W·m^−2^ for 24 h. We then examined the conversion of EGFP to tdsRed after 24 h. EGFP fluorescence was observed in all embryos, and no conversion to red fluorescence was observed (Fig. 3B). Moreover, after changing the timing of blue light illumination from E2.5 to E1.5, we did not detect red fluorescence signals by E4.5 (Fig. 3C). Thus, in B4 transgenic mice, which are expected to show consistently strong reporter fluorescence, no fluorescence conversion was detected when exposed to blue light under various conditions. This might indicate that the expression level of PA‐Cre3.0 is insufficient to induce Cre‐*loxP* recombination.

### PA‐Cre3.0 can induce light‐operated cell labeling in early embryos

**Fig. 4:**
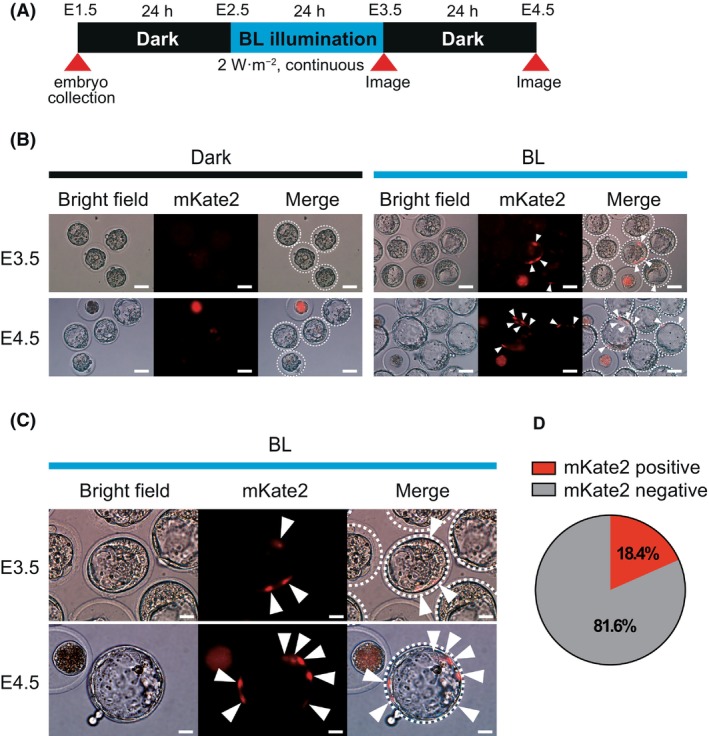
Photoactivation of PA‐Cre3.0 in homozygous Rosa^PA‐Cre A20/PA‐Cre A20^embryos. (A) Experimental protocol to confirm successful photoactivation of PA‐Cre3.0. (B) Fluorescence images of mKate2 in F2 embryos. Embryos were collected from the backcrossed between Rosa^PA‐Cre A20/WT^/CAF‐Flpe^+/WT^ F1 and R26^PA‐Cre A20/WT^ mice and cultured in the dark for 24 h, followed with or without blue light illumination for 24 h at the same conditions as in Fig. 1B. After incubation for another 24 h in the dark, embryos are observed via fluorescence microscopy. White arrowheads show mKate2 expression in the nucleus. The normal developmental embryos were circled with white dashed lines. Scale bar = 50 μm, *n* = 4. (C) High magnification image of Fig. 4B. White arrowheads show mKate2 signals in the nuclei of PA‐Cre recombinated embryos. The normal developmental embryos were circled with white dashed lines. Scale bar = 20 μm. (D) The ratio of mKate2 positive embryos in F2 mice embryos. Average of four experiments were shown. Please note that all cells in positive embryos did not exhibit mKate2 fluorescence.

As previously reported, we could confirm PA‐Cre3.0 activation with blue light and the induction of mKate2 expression after the Flp‐mediated excision of the stop signal in MEFs derived from A20 mice [12]. The lack of blue light‐operated fluorescence conversion in the embryos of F1 mice may be due to the insufficient expression of PA‐Cre3.0 and/or mKate2. Therefore, to increase PA‐Cre3.0 expression, we decided to double the copy number of PA‐Cre3.0 by homozygosing the Rosa26^PA‐Cre A20/WT^ locus in the A20 line. First, A20 heterozygous mice (Rosa26^PA‐CreA20/WT^) were bred with the CAG‐Flpe transgenic mice (CAG‐Flpe^+/WT^), and F1 mice (Rosa26^PA‐Cre A20/WT^ x CAG‐Flpe^+/WT^) were screened for hovering with both PA‐Cre3.0 at ROSA26 locus and CAG‐Flpe transgenes by genomic PCR. Then, the selected F1 mice were backcrossed with A20 heterozygous mice (Rosa26^PA‐Cre A20/WT^), which have no CAG‐Flpe transgene. F2 embryos were collected at E1.5, illuminated with blue light at 2 W·m^−2^ for 24 h from E2.5–E3.5, and observed at E4.5 (Fig. 4A). While no embryos were positive for mKate2 expression in the dark, we observed mKate2 fluorescence in the nuclei of a few F2 embryo blastomeres after blue light illumination (Fig. 4B,C). In F2 mice embryos, the theoretical probability of being homozygous at the Rosa26 locus a with the CAG‐Flpe transgene is 1/8. mKate2 fluorescence was successfully observed in 18.5% of the F2 embryos (Fig. 4D, Fig. S3). Although this efficiency is somewhat higher than the theoretical value, it is almost predictable. Furthermore, mKate2 expression in positive embryos was mosaic‐like mosaic‐like, and the proportion of positive cells varied between positive embryos. These results suggested that the doubling of the number of PA‐Cre3.0 genes by homogenization of the Rosa26 locus may have caused sufficient blue light‐dependent Cre‐*loxP* recombination, leading to the inducible expression of mKate2.

## Discussion and Conclusions

Most optogenetics‐related studies, including PA‐Cre research, have focused on cell lines or *in vivo* nervous system studies, with little work on pre‐implantation embryos. In this study, we successfully activated Cre recombinase upon exposure to blue light only by homogenizing the Rosa26 locus into which PA‐Cre3.0 was inserted. Using these light‐operated PA‐Cre3.0 mice, we demonstrated that Cre‐*loxP* recombination by blue light is possible in pre‐implantation embryos. In the A20 line with our all‐in‐one type of PA‐Cre3.0 system, blastomeres were permanently labeled with mKate2 after blue light‐mediated PA‐Cre activation. Thus, this all‐in‐one PA‐Cre3.0 system provides a new optogenetic method for analyzing early mouse development.

### PA‐Cre3.0 can be manipulated by blue light in A20 mice

No leakage of mKate2 expression in A20 mice was seen for several generations, indicating that spontaneous recombination activity in the dark was very low, consistent with previous studies [12]. This stable maintenance of mKate2 was probably due to the polyA‐stop signals inserted between *FRT* cassettes, which can be freely excised by Flp treatment. No significant differences in the survival and developmental rate of pre‐implantation embryos were observed after exposure to blue light, suggesting that the phototoxicity of 2 W·m^−2^ blue light exposure was low. After blue light illumination, mKate2 fluorescence was observed in MEFs prepared from heterozygous A20 mouse embryos. These results indicate that PA‐Cre3.0 in the A20 line functioned as originally designed; Flp can remove the polyA‐stop signals flanked by *FRT* sites and induce PA‐Cre3.0, resulting in the expression of the reporter mKate2 in a blue light‐dependent manner. Taken together, the PA‐Cre3.0 system can be safely manipulated using blue light in A20 mice.

### Homogenization promotes Cre‐ *loxP* recombination by PA‐Cre3.0

Previously, Kishi et al. [11] examined blue light‐dependent Cre‐*loxP* recombination in pre‐implantation embryos, postnatal mice, and ESCs of PA‐Cre transgenic mice. Continuous or repeated short‐pulse illumination in ESCs and postnatal animals resulted in the expression of the reporter fluorescence, while low or almost undetectable DNA recombination was observed in pre‐implantation embryos. Such differences in recombination ability with blue light also occurred in A20 mice. While previous studies using the A20 line showed high recombination rates in embryonic MEFs and the nervous system [12], the present study showed that blue light‐dependent Cre‐*loxP* DNA recombination in pre‐implantation embryos did not occur in simple crosses with CAG‐Flpe transgenic mice. An increased expression of the Cre protein or optimized light conditions are necessary for improving the recombination rate, and the usage of strong promoters has also been proposed [11].

In this study, we attempted to increase the copy number of the PA‐Cre3.0 transgene to increase the expression level of the PA‐Cre3.0 enzyme, i.e., the Cre recombination activity. To this end, we backcrossed F1 mice (A20 x CAG‐Cre transgenic mice) to A20 mice to homozygose the heterozygous A20 Rosa26 locus (Rosa^PA‐Cre A20/WT^). The results showed mosaic expression of mKate2 in a small number of F2 embryos at a genetically expected rate. This result implies that homozygosing of the Rosa26 locus with the PA‐Cre A20 transgene enabled Cre‐*loxP* recombination in a blue light‐dependent manner. We have previously shown that an increase in the expression level of PA‐Cre3.0 protein enhances recombination efficiency proportionally [12]. Thus, it might be possible that the doubling of PA‐Cre3.0 transgene copy number by homozygosing led to an increase in Cre enzyme expression levels and, consequently, to eventual mKate2 expression under blue light illumination.

Cre‐*loxP* recombination by blue light activation, detected as mKate2 fluorescence, was strongly detected in MEFs (Fig. 1) but not in heterozygous mice (Fig. 2). Also, when mKate2 was replaced by tdsRed, which exhibits stronger red fluorescence, no positive embryos could be found (Fig. 3). The reasons for this discrepancy may lie in variations in cell types and mKate2 fluorescence. Regarding differences in cell types, variations in the activity of the CAG promoter could arise between MEFs and embryos, potentially impacting the expression of PA‐Cre protein. Additionally, embryos being spherical in shape, unlike monolayer MEFs, might result in slight differences in the penetration of light into the blastomeres due to the use of a flat blue light LED panel, leading to uneven light exposure. Furthermore, embryos move during development, making it difficult to consistently illuminate blue light uniformly in the same area, leading to a decrease in the efficiency of light exposure. Consequently, the activation of PA‐Cre in embryos under the same illumination conditions as MEFs might be insufficient, necessitating a detailed optimization of light exposure. Additionally, mKate2 is a far‐red fluorescent protein with reduced biological toxicity compared to tdsRed, but it is characterized by low brightness [20]. This aspect likely contributed to challenges in its detection. Thus, the lack of quantitative analysis and the failure to examine various blue light illumination conditions can be considered a limitation of this study, and further improvements required for next step.

### Comparison with previous reports and future perspectives

In previous studies, blue light‐dependent Cre‐*loxP* recombination by PA‐Cre has been detected, but at a very low frequency [11]. In contrast, Cre‐*loxP* recombination‐mediated mKate2 expression was typically observed in A20 mice. There are two possible reasons for this discrepancy. First, A20 mice have unique feature on PA‐Cre expression at the Rosa26 locus, which is an all‐in‐one type of PA‐Cre3.0 expression system carrying the fluorescent protein mKate2 as a recombination detection reporter. PA‐Cre3.0 is an improved version of the first generation of PA‐Cre reported in a previous study [12]. The A20 system is also equipped with a suicide option, in which blue light‐activated PA‐Cre3.0 causes self‐recombination to remove PA‐Cre3.0 interposed by *loxP* sites. Because the irreversible and continuous expression of Cre exerts toxic effects on cells and tissues [21], its transient activation may have resulted in the successful observation of mKate2‐positive embryos through reduced Cre toxicity. Furthermore, the reporter mKate2 is on the same vector as PA‐Cre3.0; hence, the homozygous status of both genes can be easily adjusted by a single cross and maintained through easy breeding.

With recent advances in red light‐induced PA‐Cre and other photoactivatable recombination systems, such as Flp‐*FRT* [5, 22, 23], these new light‐operated recombination systems will enable noninvasive, spatiotemporal, and cell‐specific DNA recombination in pre‐implantation embryos, providing a tool that can be applied to a wide range of biomedical studies, including the precise analysis of cell lineage and gene function.

## Conflict of interest

The authors declare no conflict of interest.

### Peer review

The peer review history for this article is available at https://www.webofscience.com/api/gateway/wos/peer‐review/10.1002/2211‐5463.13862.

## Author contributions

KM and YS conceived and designed the project, KM, AN, and LS acquired the data, KM, AN, LS and TE analyzed and interpreted the data, KM, EK, and YS wrote the paper.

## Supporting information


**Fig. S1.** Schematic representation of the PA‐Cre 3.0 system.
**Fig. S2.** PCR analysis of the genomic DNA of MEFs derived from A20 fetuses.
**Fig. S3.** Number of mKate2 positive embryos in F2 mice embryos.

## Data Availability

The data that support the findings of this study are available from the corresponding author, Kumi Morikawa [kumi-morikawa@aist.go.jp] upon reasonable request.
