# Supplementary material for: Optogenetic control of early embryos labeling using photoactivatable Cre recombinase 3.0
Source: FEBS Open Bio. 2024 Sep 2;14(11):1888–98. doi: 10.1002/2211-5463.13862 (PMC11532978; doi:10.1002/2211-5463.13862)
Supplement: Supplementary file 1 — Fig. S1. Schematic representation of the PA‐Cre 3.0 system. Fig. S2. PCR analysis of the genomic DNA of MEFs derived from A20 fetuses. Fig. S3. Number of mKate2 positive embryos in F2 mice embryos. [file FEB4-14-1888-s001.docx]

**
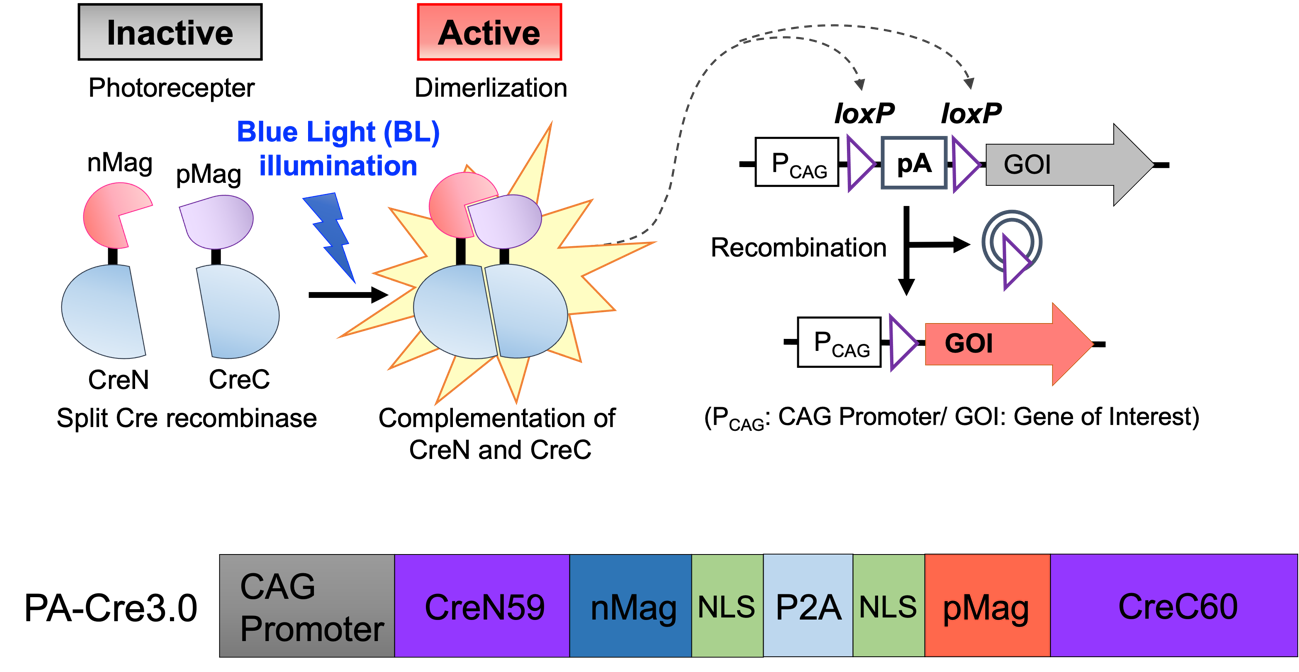
**

**Fig. S1 Schematic representation of the PA-Cre 3.0 system.** PA-Cre3.0 comprises two split Cre fragments, CreN and CreC. Upon blue light illumination, the two split Cres fragments are complemented along with the light-dependent dimerization of nMag-pMag to achieve catalytic activity for *loxP* site recombination.

**Fig. S2 PCR analysis of the genomic DNA of MEFs derived from A20 fetuses.** PCR analysis of genomic DNA from wild-type mice (lane 1) and A20:CAG-Flp F1 fetuses (lanes 2–9). Primers for PCR analysis are listed in the Materials and Methods section. Upper panel: with Rosa^PA-Cre A20^ primers, middle panel: with CAG-Flpe primers, lower panel: with primers to detect stop sequence excision, in which the intact and the stop excision loci generate 2964- and 415-bp bands, respectively.

**Fig. S3 Number of mKate2 positive embryos in F2 mice embryos.** 24hr after blue lights illumination, mKate2 positive embryos were counted in four separate experiments.
